# Nurses’ Roles in Supporting Digital Engagement and Self-Management in Adults with Type 2 Diabetes: A Scoping Review

**DOI:** 10.3390/nursrep16060191

**Published:** 2026-06-04

**Authors:** Jalal Uddin, Tazveen Fariha, Shahida Sultana Shumi, Farzana Rahman, Md Ariful Islam, Susmita Saha Proma, Bishwajit Sarker

**Affiliations:** 1 Department of Epidemiology and Biostatistics, University of Nevada, Las Vegas, Las Vegas, NV 89154, USA; 2 Department of Social and Behavioral Health, University of Nevada, Las Vegas, Las Vegas, NV 89154, USA; fariha@unlv.nevada.edu; 3 Health Knowledge and Research Foundation (HKRF), Chattogram 4366, Bangladesh; shumi@hkrfhealth.org; 4 Department of Physiotherapy, Centre for the Rehabilitation of the Paralysed (CRP), Mirpur-14, Savar, Dhaka 1216, Bangladesh; min.far28@gmail.com (F.R.); physiosusmitasaha@gmail.com (S.S.P.); 5 Medical Centre, Gazipur Agricultural University, Gazipur 1706, Bangladesh; arifphysio.bsmrau@gmail.com; 6 Department of Physiotherapy and Rehabilitation, Enam Medical College and Hospital, 9/3 Parbotinagar, Thana Road, Savar 1340, Bangladesh; physiobishwajitsarker@gmail.com

**Keywords:** type 2 diabetes, nurses, digital engagement, digital health literacy, self-management, telehealth, patient portals, scoping review

## Abstract

**Background**: Adults with type 2 diabetes increasingly use patient portals, telemonitoring systems, mobile applications, text messaging programs, and other digital services to support self-management. In practice, however, these approaches often still depend on nursing support to help patients understand, use, and sustain digital care in everyday settings. This scoping review mapped how nurses are involved in supporting adults with type 2 diabetes to use digital tools, information, and services for self-management across care settings. **Methods**: This scoping review followed Joanna Briggs Institute methodology and was reported in line with the Preferred Reporting Items for Systematic Reviews and Meta-Analyses extension for Scoping Reviews. The review question was guided by the Population, Concept, and Context framework. A literature search was conducted in January 2026 in PubMed, Scopus, Embase, and EBSCO/CINAHL. A total of 230 records were identified, 71 duplicates were removed, and 159 records underwent title and abstract screening. Fifty-three full-text articles were assessed for eligibility, and 15 studies met the inclusion criteria. Data were extracted using a structured charting table and synthesized descriptively and thematically. **Results**: The 15 included studies were published between 2021 and 2026 and represented evidence from 10 countries across primary care, community health centers, telehealth programs, and hospital-linked services. Five interrelated themes were identified: nurses as digital self-management educators; nurses as remote monitors and care coordinators; nurses as facilitators of digital engagement, confidence, and supported use; nurses as implementation partners in digital diabetes care; and equity, access, and context as shaping conditions of digital diabetes support. Only one study directly measured digital health literacy, whereas the remaining studies addressed digital engagement more indirectly through onboarding, portal communication, telemonitoring, reminders, tailored feedback, and implementation work. Common barriers included workload, unclear responsibilities, technical difficulties, age- or literacy-related access challenges, language needs, and uneven infrastructure. **Conclusions**: The included studies suggest that nurses commonly contributed to making digital diabetes care more understandable, usable, and actionable for adults with type 2 diabetes. Their roles were described across education, monitoring, coordination, implementation, and support for digital engagement. Future studies could measure digital health literacy more explicitly, describe nursing tasks in greater detail, and examine how equity-related factors shape digital diabetes care.

## 1. Introduction

Type 2 diabetes mellitus is a long-term condition that depends heavily on daily self-management. For many adults, this includes regular attention to medication use, blood glucose monitoring, diet, physical activity, symptom recognition, and follow-up care [1]. In recent years, digital approaches to diabetes care have expanded quickly and now include patient portals, telemonitoring systems, mobile health applications, text messaging programs, virtual assistants, and web-based self-management platforms [2,3,4,5]. These models are often described as ways to improve access, strengthen communication, and support self-management in more flexible and timely ways.

At the same time, digital care does not automatically become meaningful simply because a tool is available. For many patients, using digital services still requires help with interpretation, confidence, continuity, and application to daily life [6,7]. A portal message, reminder, or transmitted glucose value may have limited practical value unless the patient understands what it means and what to do next. This is especially important in type 2 diabetes, where self-management depends on routine decisions about medication, monitoring, food, activity, and follow-up outside formal clinic visits [1]. In that sense, digital systems are not separate from care relationships; they become useful through the support structures around them.

A growing body of literature suggests that digital interventions can improve aspects of diabetes care, including glycemic outcomes and self-management, although the size and consistency of benefit vary across intervention types and settings [8,9]. Effective programs often combine technology with structured follow-up, feedback, and ongoing professional support rather than relying on technology alone [2,10]. This makes the role of nurses especially relevant. In routine and digitally supported diabetes care, nurses may educate patients, reinforce treatment plans, monitor clinical changes, respond between visits, support portal or app use, interpret digital feedback, and help services integrate digital programs into everyday workflow [11,12,13,14].

Another issue is that the language used in this field is not always consistent. Some studies refer directly to digital health literacy or electronic health literacy, while others describe related ideas through supported portal use, confidence in technology, self-tracking, digital communication, or help with navigation and interpretation [5]. Digital health literacy is clearly important, but it may not fully capture the wider set of nursing activities involved when patients are helped to begin using digital tools, continue using them, and connect them to practical self-management [7]. For that reason, digital engagement may be a more useful organizing concept in this context. It allows room for digital health literacy where it is measured directly, while also recognizing the broader support that nurses provide when helping patients use digital tools, information, and services in everyday care.

The public health importance of this issue is also clear. Digital diabetes care does not work in the same way for all patients. Age, language, literacy, prior digital experience, access to devices, internet connectivity, and local service resources can shape whether digital support is realistic and useful in practice [5,15,16]. As digital models become more common in chronic disease care, it becomes increasingly important to understand not only whether digital services are available, but also how they are implemented in practice, who benefits from them, and what role nurses play in making them work [10,11,14].

Although previous studies and reviews have examined digital diabetes interventions, telehealth, mobile health, patient portals, and self-management outcomes, the specific contribution of nurses within these digital care models remains insufficiently synthesized. Existing evidence is dispersed across different technologies, care settings, study designs, and outcome types. Some studies emphasize glycemic outcomes, others focus on implementation or patient engagement, and many describe nurses only as part of the intervention team without clearly explaining their practical role. As a result, it remains unclear how nurses support adults with type 2 diabetes in adopting, using, interpreting, and sustaining digital tools for self-management across routine care settings. This gap is important because digital diabetes care often depends on human support, workflow integration, and patient-centered guidance rather than technology alone.

This scoping review addresses this gap by mapping how nurses are involved in supporting digital engagement and self-management among adults with type 2 diabetes across diverse digital care models and settings. The novel contribution of this review is its focus on nurses’ practical roles in digital diabetes care, including education, remote monitoring, care coordination, digital onboarding, implementation support, and equity-related support for patients with varying levels of access, literacy, and digital confidence. The review question was: How are nurses involved in supporting adults with type 2 diabetes to use digital tools, information, and services for self-management across care settings?

## 2. Materials and Methods

This study adopted a scoping review design to map the existing literature on nurses’ roles in supporting digital engagement and self-management among adults with type 2 diabetes. A scoping review was appropriate because the literature spans multiple digital modalities, clinical settings, nursing roles, and study designs, and the aim was to describe the scope, characteristics, and key concepts of the evidence rather than to estimate pooled intervention effects. The review was conducted in accordance with the Joanna Briggs Institute (JBI) methodology [17,18] and reported according to the Preferred Reporting Items for Systematic Reviews and Meta-Analyses Extension for Scoping Reviews (PRISMA-ScR) [19]. The review protocol was registered with the Open Science Framework (OSF) and is available at: https://osf.io/a6582/overview (accessed 4 April 2026).

### 2.1. Eligibility Criteria

The inclusion criteria were defined using the Population, Concept, and Context (PCC) framework.

Population: Adults aged 18 years or older with type 2 diabetes mellitus.

Concept: Nurses’ roles in supporting patients’ use of digital tools, information, and services for diabetes self-management. This included nursing support delivered through telemonitoring, patient portals, text messaging, mobile health systems, web-based platforms, virtual assistants, app-based programs, and other digital models relevant to self-management.

Context: Primary care, community health centers, outpatient services, telehealth programs, hospital-linked diabetes services, and other clinical or community settings in which adults with type 2 diabetes received digitally supported care.

Studies were eligible if they met the following criteria: (1) focused on adults with type 2 diabetes; (2) described a nursing role directly connected to a digital intervention, digital service, or digitally supported self-management process; (3) included a meaningful digital component relevant to diabetes self-management; (4) reported findings related to self-management, medication adherence, monitoring, education, care coordination, implementation, or patient engagement; and (5) were published in English and available in full text. Quantitative, qualitative, mixed-methods, pilot, observational, implementation, and randomized studies were eligible. Grey literature, dissertations, conference abstracts, editorials, commentaries, and protocols were excluded because this review aimed to synthesize peer-reviewed empirical studies with sufficient methodological detail and extractable findings on nurses’ roles in digital diabetes care. The search was restricted to English-language full-text articles because of the review team’s language capacity and to ensure accurate screening, data extraction, and interpretation of intervention details. We recognize that this approach may have excluded relevant non-English studies and unpublished implementation evidence.

### 2.2. Search Strategy

The review question was guided by the Population, Concept, and Context (PCC) framework for scoping reviews. In this review, the population was adults with type 2 diabetes, the concept was nursing support for digital engagement and self-management, and the context was digitally supported diabetes care across clinical and community settings. A literature search was conducted in January 2026 using four electronic databases: PubMed, Scopus, Embase, and EBSCO/CINAHL. The search was designed to identify studies examining nurses’ roles in supporting adults with type 2 diabetes in the use of digital tools, information, and services for self-management. The following search terms were used: (“type 2 diabetes” OR T2DM) AND (nurs* OR “nurse-led” OR “nursing intervention*” OR “diabetes nurse*” OR “nurse educator*”) AND (“digital health literacy” OR “eHealth literacy” OR “electronic health literacy” OR “mHealth literacy” OR “digital literacy” OR telehealth OR telemedicine OR mHealth OR eHealth OR “mobile app*” OR “patient portal*” OR “online health information” OR “web-based”) AND (“self-management” OR “self care”). Search strategies were adapted as appropriate for the syntax and indexing structure of each database. The search yielded 230 records in total, including 58 from PubMed, 69 from Scopus, 99 from Embase, and 4 from EBSCO/CINAHL. Key search concepts included type 2 diabetes, nursing roles, digital health or digital service modalities, and self-management. The complete database-specific search strategies, including syntax adaptations, publication-year handling, and search yields, are provided in Supplementary Table S1. The relatively small number of records retrieved from EBSCO/CINAHL likely reflected the specificity of the search strategy, which required simultaneous reference to type 2 diabetes, nursing roles, digital health or digital service terminology, and self-management. In addition, relevant nursing studies may have been indexed under broader diabetes education, telehealth, or chronic disease management terms without all search concepts appearing together.

### 2.3. Study Selection

All identified records were uploaded to Rayyan (Rayyan Systems Inc., Cambridge, MA, USA) to support blinded screening and duplicate removal. Optional AI-assisted features in Rayyan were not used. Screening and eligibility assessment were conducted manually and independently by two reviewers, Biswajit Sarker and Farzana Rahman, with independent oversight by Jalal Uddin. Prior to full screening, a pilot test was conducted on the first 50 records to assess consistency in interpretation of the eligibility criteria. Agreement exceeded 90%, and any discrepancies were discussed before proceeding with full screening.

Of the 230 records identified, 71 duplicates were removed. The remaining 159 unique records underwent title and abstract screening. At this stage, 106 records were excluded. A total of 53 full-text articles were assessed for eligibility, and 38 were excluded after full-text review. Fifteen studies met the inclusion criteria and were retained in the final review. Disagreements during title/abstract screening and full-text eligibility assessment were first resolved through discussion between the two reviewers (FR and BS). When consensus could not be reached, the record was reviewed by a third reviewer (JU), who verified the eligibility criteria and supported the final decision. Reasons for exclusion at the full-text stage were recorded. The overall study selection process is presented in a PRISMA-ScR flow diagram.

### 2.4. Data Extraction

Data were extracted using a structured charting table aligned with the review objectives and PCC framework. The extracted information included title, authors, year of publication, country, care setting, study design, participant characteristics, digital intervention or platform, description of the nursing role, self-management focus, and key findings relevant to the review question. Additional information was charted when available regarding digital engagement, digital health literacy, implementation issues, and reported barriers or facilitators.

Data extraction was conducted independently by two reviewers (FR and BS), and discrepancies were resolved through discussion and verification by a third reviewer (JU). During the initial data extraction phase, agreement between reviewers exceeded 95%. When needed, extracted information was checked against the full text to improve accuracy and completeness.

### 2.5. Variables of Interest

#### 2.5.1. Factors Examined Across Studies

In this scoping review, variables were extracted as they were examined in the included studies. Consistent with scoping review methodology, these variables were not treated as fixed independent or dependent variables across all studies; rather, they were considered study-specific factors whose role varied according to study design, setting, and analytic approach.

The most commonly examined factors included patient age, prior digital experience, digital confidence, socioeconomic position, language needs, access to devices or internet connectivity, care setting, workflow integration, staffing capacity, and implementation support. Several studies also addressed contextual factors that influenced uptake, engagement, and sustainability of digital diabetes care.

#### 2.5.2. Outcomes of Interest

The primary outcome of interest was the role of nurses in supporting digital engagement for self-management among adults with type 2 diabetes. Broadly, this included how nurses helped patients understand, adopt, navigate, interpret, and sustain the use of digital tools, information, and services relevant to routine diabetes care.

Secondary outcomes included glycemic indicators, self-management behaviors, medication adherence, self-efficacy, engagement with digital systems, acceptability, usability, implementation experiences, and contextual barriers and facilitators. Because these outcomes were measured heterogeneously across studies, they were synthesized descriptively rather than quantitatively.

#### 2.5.3. Classification of Digital Health Literacy and Digital Engagement

In this review, digital health literacy was understood as patients’ ability to access, understand, appraise, and use digital health information or services to support diabetes-related decisions and self-management. This included direct measurement using digital or eHealth literacy items or scales, as well as study descriptions of patients’ ability to use portals, apps, telemonitoring systems, or digital communication tools for diabetes care.

Digital engagement was used as a broader organizing concept. We defined digital engagement as patients’ practical involvement with digital diabetes tools and services over time, including initial access, onboarding, navigation, use of digital functions, interpretation of digital feedback, communication with providers, and sustained application of digital information to self-management. Unlike digital health literacy, which emphasizes skills and capacity, digital engagement also captures behavior, support, confidence, continuity, and the care processes that help patients use technology in practice. Digital engagement is therefore related to, but distinct from, technology adoption, trust in digital tools, adherence, and self-efficacy. In this review, direct and indirect indicators related to digital engagement were extracted and summarized as reported by the original study authors, without reclassifying them into a single standardized metric.

### 2.6. Data Synthesis

First, the characteristics of the included studies were summarized descriptively, including year of publication, country, care setting, study design, digital modality, and broad nursing role. Second, a thematic synthesis was conducted to identify recurring patterns in how nurses supported adults with type 2 diabetes in using digital tools, information, and services for self-management. Through repeated reading and comparison of the extracted data, studies were grouped according to similarities and differences in nursing roles, patient support processes, implementation concerns, and contextual influences.

The final synthesis was organized into five themes: nurses as digital self-management educators; nurses as remote monitors and care coordinators; nurses as facilitators of digital engagement, confidence, and supported use; nurses as implementation partners in digital diabetes care; and equity, access, and context as shaping conditions of digital diabetes support. Digital engagement was treated as the broader organizing concept because many studies described nurses helping patients use digital systems in practical ways without explicitly labeling those processes as digital health literacy. Findings from the methodological quality appraisal were used to contextualize the thematic synthesis and to identify which conclusions were supported by stronger or more limited evidence.

### 2.7. Methodological Quality Appraisal

Although formal critical appraisal is not always required for scoping reviews, we conducted a methodological quality appraisal to support interpretation of the evidence. The Mixed Methods Appraisal Tool (MMAT), version 2018 [20], was used because it is designed to appraise qualitative, quantitative randomized, quantitative non-randomized, quantitative descriptive, and mixed-methods studies. Two reviewers independently appraised each included study using the MMAT category appropriate to its design. Discrepancies were resolved through discussion, with consultation from a third reviewer when needed. The appraisal was used to contextualize the strength and limitations of the evidence, not to exclude studies from the review.

### 2.8. Review Team and Rigor

To strengthen methodological rigor, screening and data extraction were conducted independently by more than one reviewer, disagreements were resolved through discussion, and a pilot screening exercise was completed before full screening. The use of the PCC framework, PRISMA-ScR reporting structure, structured data charting, and iterative thematic synthesis further supported transparency and consistency across the review process.

## 3. Results

### 3.1. Study Identification and Selection

**Figure 1:**
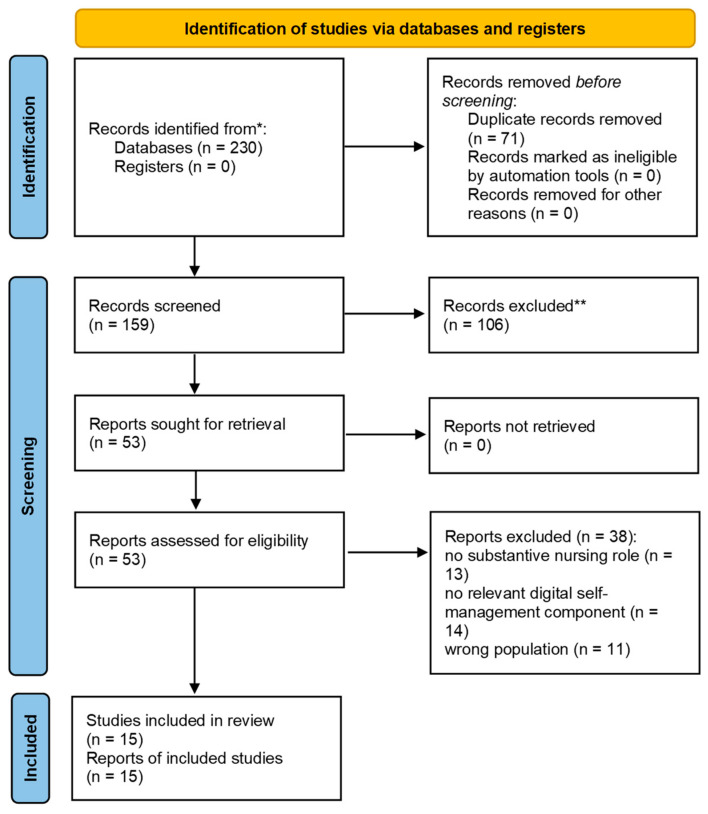
PRISMA-ScR flow diagram of study selection. * Records identified from electronic databases (PubMed, Scopus, Embase, and EBSCO/CINAHL). ** Records excluded during title and abstract screening.

The database search identified 230 records from four sources: PubMed (*n* = 58), Scopus (*n* = 69), Embase (*n* = 99), and EBSCO/CINAHL (*n* = 4). After removal of 71 duplicate records, 159 unique records remained for title and abstract screening. Of these, 106 records were excluded. Fifty-three full-text articles were assessed for eligibility, and 38 were excluded after full-text review. Fifteen studies met the inclusion criteria and were included in the final synthesis. The study selection process is summarized in Figure 1.

### 3.2. Characteristics of Included Studies

The 15 included studies were published between 2021 and 2026 and represented a geographically diverse body of evidence from Denmark, the United Kingdom, Spain, the United States, South Africa, China, Sweden, Singapore, Malaysia, and the Netherlands. Most studies were conducted in routine care environments, including general practice, primary care, community health centers, telehealth services, and hospital-linked diabetes programs. The included evidence comprised qualitative studies, mixed-methods and implementation studies, pilot and observational studies, and randomized or prospective intervention designs.

Digital care models varied across studies and included telemonitoring systems, text message-based adherence interventions, patient portal support, mobile health management systems, app-enabled insulin titration and chronic disease management programs, web-based risk assessment platforms, and virtual assistants delivered through messaging applications. Despite this variation, the nursing role was rarely confined to a single function. Instead, nurses contributed across multiple domains, including patient education, remote monitoring, care coordination, digital onboarding, motivational support, and implementation work. Table 1 summarizes the characteristics of the included studies.

### 3.3. Methodological Quality of Included Studies

Methodological quality varied across the included studies. Stronger evidence came from studies with clearer designs, well-described participant samples, appropriate data collection methods, and transparent reporting of intervention components or implementation processes. Randomized, prospective, and larger observational studies contributed more strongly to understanding clinical and self-management outcomes, whereas qualitative and implementation studies provided richer insight into workflow, role clarity, feasibility, equity, and contextual barriers. Common methodological limitations included small sample sizes, single-site designs, limited follow-up, incomplete reporting of nursing workload, and inconsistent measurement of digital health literacy or digital engagement. Because of this variation, findings were interpreted according to the relative strength and purpose of each study rather than treating all included studies as carrying equal evidentiary weight. A detailed summary of the methodological quality appraisal is provided in Supplementary Table S2.

### 3.4. Thematic Synthesis

Five interrelated themes were identified from the included studies: (1) nurses as digital self-management educators; (2) nurses as remote monitors and care coordinators; (3) nurses as facilitators of digital engagement, confidence, and supported use; (4) nurses as implementation partners in digital diabetes care; and (5) equity, access, and context as shaping conditions of digital diabetes support. Overall, the findings showed that nurses played a central role in making digital diabetes care understandable, usable, and actionable in routine practice.

#### 3.4.1. Nurses as Digital Self-Management Educators

Across the included studies, nurses frequently acted as educators who translated digital systems into practical self-management tasks. Their role extended beyond providing diabetes information and included helping patients understand how to use digital tools in relation to glucose monitoring, medication routines, insulin management, diet, physical activity, and follow-up care. In telehealth and mobile health interventions, nurses commonly delivered structured education at enrollment and reinforced diabetes knowledge during follow-up. In portal-based and app-supported models, they helped patients connect digital reminders, communication, and self-monitoring functions with daily self-management behaviors. In qualitative and implementation-oriented studies, educational work also included helping patients understand the purpose of the technology, how it fit into care, and why continued engagement mattered.

#### 3.4.2. Nurses as Remote Monitors and Care Coordinators

A second major theme was the role of nurses as remote monitors and care coordinators. Several studies described nurses as the clinicians who reviewed transmitted data, responded to abnormal readings, reinforced treatment plans, and linked digital information with ongoing clinical follow-up. This role was especially visible in telemonitoring, insulin-management, and chronic disease monitoring models, where nurses maintained continuity between scheduled visits and supported patients in responding to threshold alerts, symptom changes, or self-reported data. In these studies, digital systems did not function as stand-alone technologies; rather, they became clinically meaningful because nurses interpreted incoming information, initiated follow-up, and sustained care coordination over time.

#### 3.4.3. Nurses as Facilitators of Digital Engagement, Confidence, and Supported Use

The included studies also showed that nurses were central to helping patients begin, sustain, and benefit from digital care. This role included onboarding, individualized portal or app training, reinforcement of confidence, interpretation of digital messages or feedback, and ongoing encouragement to continue using digital systems. Only one study directly measured digital health literacy; Whittemore et al. assessed digital health literacy using four self-report items scored on a 1–5 agreement scale, including items on independent use of apps and ability to solve basic technical issues [5]. In the remaining studies, digital engagement was addressed more indirectly through supported use of portals, telemonitoring systems, reminders, self-tracking tools, and digital communication platforms. Taken together, these studies suggest that nurses often support a practical form of digital engagement in routine care even when that work is not explicitly described as digital health literacy.

#### 3.4.4. Nurses as Implementation Partners in Digital Diabetes Care

Many of the included studies contributed not only to understanding patient support, but also to understanding how nurses help digital interventions become workable within routine services. In implementation-focused and qualitative studies, nurses played important roles in shaping content, refining workflows, identifying barriers, tailoring interventions to local contexts, and determining how digital programs could fit existing care pathways. Recurrent implementation concerns included role clarity, staffing capacity, workflow compatibility, message timing, referral processes, onboarding demands, and technical usability. These findings indicate that nurses were not simply end users of digital diabetes interventions; they were also active implementation partners whose practical knowledge influenced whether digital programs could be successfully integrated into routine care.

#### 3.4.5. Equity, Access, and Context Shape Digital Diabetes Support

The final theme concerned equity, access, and context. Across the included literature, digital diabetes support was strongly influenced by patient- and service-level conditions such as age, prior digital experience, language, literacy, socioeconomic position, connectivity, staffing, and available infrastructure. In studies involving underserved populations, including low-income and portal-naive patients, access support and confidence-building were central to intervention delivery. Other studies highlighted language needs, transport constraints, variable digital familiarity, and concerns that digital programs might exclude patients with limited resources or low digital confidence unless additional support was intentionally built in. These findings indicate that nursing support for digital diabetes care is shaped not only by the technology itself, but also by broader contextual and equity-related conditions. The themes identified across the included studies are summarized in Table 2.

Taken together, the included studies indicate that nurses play a broad and multi-layered role in digitally supported type 2 diabetes care. Their contribution extends beyond teaching or troubleshooting and includes monitoring, coordination, implementation work, and sustained support for patients’ use of digital systems. The evidence base more strongly supports a concept of nursing support for digital engagement than a narrow concept of digital health literacy alone, as explicit measurement of digital health literacy was uncommon. Overall, the findings show that digital diabetes care remains heavily dependent on nursing work to translate technologies into practical self-management support across diverse care settings.

## 4. Discussion

This scoping review suggests that nurses play a steady, practical role in helping adults with type 2 diabetes use digital tools and services in ways that support everyday self-management. Across the 15 included studies, nurses were involved not only as educators, but also as remote monitors, care coordinators, facilitators of ongoing digital use, and contributors to implementation. Taken together, the findings suggest that nursing support in this area is broader than digital health literacy alone. In many settings, nurses were helping patients make digital information, alerts, messages, and monitoring systems usable in daily life rather than simply introducing the technology itself [2,5,11].

One of the clearest patterns across the review was that digital support and self-management support were closely linked. In telehealth and remote monitoring studies, nurses were not simply checking incoming data. They helped patients understand what the readings meant, connected those data to treatment plans, reinforced medication and lifestyle routines, and supported decisions between visits [2,13,24,25]. In portal-, app-, and messaging-based interventions, nurses also helped patients begin using the system, continue using it over time, and relate digital tasks to everyday activities such as medication use, diet, exercise, insulin management, and follow-up care [3,5,22].

This pattern is in line with the broader diabetes literature. A recent systematic review and meta-analysis of digital interventions for type 2 diabetes found that these interventions can improve glycemic outcomes, but results vary across intervention types and study designs. Notably, one of the more consistent features of successful interventions was the presence of timely, responsive coaching from a health professional rather than technology alone [8]. A more recent meta-analysis focused specifically on nurse-led digitalized diabetes management programs also found improvements in self-care, quality of life, and HbA1c, again pointing to the value of ongoing professional support alongside digital tools [9]. Together, these findings support the idea that digital systems are most useful when they are part of a supported care process rather than a stand-alone technical solution.

Another important finding from this review was the visibility of nurses as implementation actors. Several of the included studies, especially qualitative and implementation-focused papers, showed nurses helping to shape referral pathways, message timing, onboarding processes, workflow integration, and the practical conditions needed for digital interventions to function in routine care [11,12,14,21,23]. This matters because digital diabetes care is not introduced into empty space. It has to fit within existing services, staff roles, time constraints, and patient needs. A recent scoping review of nurse-led telehealth and mobile health models in low-income US populations reached a similar conclusion, noting that technology, communication, and community support worked together rather than independently in shaping patient outcomes [10]. In practice, this means that whether a digital program works may depend as much on service design and workflow as on the technical features of the intervention itself.

A conceptual synthesis across the included studies suggests that nurses’ roles varied according to the dominant function of the digital intervention and the care context in which it was implemented. In telemonitoring and insulin-support models, nursing work was more clinically oriented and included reviewing patient-generated data, responding to abnormal readings, supporting medication-related decisions, and coordinating follow-up [2,13,21,22]. In portal-, app-, and messaging-based interventions, nurses more often functioned as digital engagement facilitators by helping patients access systems, interpret messages, maintain confidence, and connect digital tasks to daily self-management [3,5,25]. In qualitative and implementation-focused studies, nurses were positioned as workflow and implementation partners whose practical knowledge shaped whether digital programs could be integrated into routine care [2,13,24,25]. Taken together, these patterns suggest a technology–nursing work–self-management pathway: digital tools generate information, prompts, or communication opportunities; nurses translate, monitor, personalize, and coordinate these digital inputs; and patients use the supported information to guide everyday diabetes self-management. This model helps explain why digital tools alone may be insufficient without nursing support and why nursing roles differ across intervention types, patient populations, and health system settings.

The review also showed that digital health literacy was rarely measured directly. Only one included study explicitly assessed digital health literacy [5]. Most of the other studies referred instead to related processes, such as supported portal use, reminders, confidence in using digital tools, self-tracking, interpretation of readings, or help with navigating technology. This does not weaken those studies. Rather, it suggests that nursing support for digital engagement is already happening in practice, even if it is not always described in the same terms. A 2024 study of adults with type 2 diabetes found that electronic health literacy was related to self-management through self-efficacy and social support, which suggests that digital understanding is part of a wider self-management process rather than an isolated skill [26]. This pathway is consistent with the present review because nursing support may strengthen digital engagement through similar mechanisms: building patients’ confidence to use digital systems, providing social and professional reinforcement, and helping patients translate digital information into self-management actions. Thus, digital engagement should be understood not only as technology use, but also as a supported process shaped by self-efficacy, relational support, and care continuity.

For that reason, digital engagement appears to be the more useful organizing concept for this review. It allows room for formal digital health literacy where it is measured, but it also captures the broader work that nurses were doing across the included studies: helping patients start using digital systems, keep using them, interpret what they saw, and apply that information in daily care. That framing seems especially appropriate here because the nursing role often involved more than knowledge transfer. It also involved encouragement, translation, troubleshooting, reinforcement, and continuity.

The findings also point to the public health importance of context. Many of the included studies were carried out in primary care, community health centers, and routine chronic disease services rather than in highly controlled pilot settings. In those environments, nurses often became the people who noticed when age, low literacy, language needs, limited resources, low digital confidence, or weak technical infrastructure affected participation in digital care [4,5,15,16,22]. This issue goes beyond the present review. A 2024 interview study in community health centers found that both patients and clinicians continued to report substantial barriers to patient portal use, including limited health and technology literacy, trouble maintaining engagement, and concern about clinician burden [6]. A 2025 systematic review and meta-analysis on digital health literacy in people with common chronic diseases similarly noted that age, education, employment, and views of the internet as a health resource were associated with digital health literacy, while the limited number of studies constrained deeper analysis of determinants and outcomes [27]. These findings suggest that equity should not be treated as a side issue in digital diabetes care. It is one of the main factors that shapes whether digital support is realistic and useful in practice.

These findings also raise an important question about whether digital diabetes care represents an expansion of the nursing role, a substitution for traditionally physician-led functions, or a redistribution of work across the care team. In several studies, nurses were not only reinforcing education but also reviewing digital data, responding to abnormal readings, supporting medication-related decisions, coordinating follow-up, and helping patients interpret technology-generated feedback [2,13,24,25]. These activities suggest that digital care may expand nursing responsibilities beyond conventional education and follow-up, particularly in telemonitoring and insulin-support models. However, this expansion should not be interpreted as simple task substitution unless clinical governance, escalation pathways, role boundaries, and training requirements are clearly defined. Without these supports, digital programs may increase nursing workload while leaving responsibility, time allocation, and accountability insufficiently formalized.

The feasibility of these expanded roles depends on staffing capacity, protected time, digital training, technical support, and integration into routine workflows. Digital interventions are sometimes expected to reduce service burden, but the included studies suggest that technology can also create new work, including onboarding patients, monitoring incoming data, troubleshooting access problems, responding to alerts, and sustaining engagement [11,14,20,24]. Therefore, implementation should include explicit planning for nursing workload, documentation procedures, escalation protocols, and staffing models. Programs that rely on nurses to make digital diabetes care usable should recognize this work as a core component of care delivery rather than an informal extension of existing duties.

This review also has implications for nursing practice. If digital diabetes care is to become part of routine services, then nursing time, training, and workload need to be considered explicitly. The studies included here suggest that nurses often take on tasks such as onboarding, remote follow-up, motivational support, and interpretation of digital feedback, yet these tasks are not always described in enough detail to show how much work they involve or how they affect routine care. The broader literature points in the same direction. Nurse-led digitalized programs appear promising, but their value seems tied to structured support, repeated contact, and meaningful follow-up rather than technology alone [9,10]. From a service perspective, that means digital care should not automatically be assumed to reduce labor. In some cases, it may shift work rather than reduce it, with nurses taking on responsibilities that need formal recognition and support.

There are also implications for education and future research. In education, skills related to telemonitoring interpretation, digital communication, portal support, and technology-enabled coaching may need to be treated as part of core nursing preparation rather than optional additions. In research, more precise reporting is needed on what nurses actually do in digital diabetes interventions, how much time these activities require, what training is provided, and how tasks are shared across team members. Future studies would also benefit from clearer distinctions among digital health literacy, digital confidence, supported use, access support, and digitally mediated self-management behaviors. Without that clarity, it will remain difficult to compare studies or to understand which parts of digital care are actually driving outcomes.

When interpreted in light of methodological quality, the strongest conclusions from this review concern the breadth of nursing roles across digital diabetes care and the importance of human support in making digital tools actionable (Table 1; Supplementary Table S2). These conclusions were supported across multiple study designs and settings. Evidence regarding clinical outcomes such as glycemic control, medication adherence, or sustained self-management improvement should be interpreted more cautiously because outcome measures, follow-up periods, and intervention designs varied substantially [2,4,13,16,21]. Qualitative and implementation studies were especially valuable for understanding feasibility, workflow, role clarity, and contextual barriers, but they were less suited to determining effectiveness [11,12,14,20,22,24,25]. Therefore, the synthesis supports the practical importance of nursing roles in digital engagement while also highlighting the need for stronger, more consistently reported intervention studies.

In summary, this review indicates that digitally supported diabetes care remains strongly dependent on human support. Across the included studies, nurses helped make digital tools understandable, workable, and sustainable by translating digital information into practical self-management support. This contribution should be described more explicitly in future research and deliberately supported in practice.

### Strengths and Limitations

This review has several strengths. It followed a structured scoping review approach, used clear eligibility criteria based on the Population–Concept–Context framework, and applied independent screening and data extraction procedures to strengthen rigor and consistency. The review also included studies from multiple countries and care settings, which provided a broader view of how nurses support digitally mediated self-management among adults with type 2 diabetes. In addition, full-text review of all included studies supported a more detailed and accurate thematic synthesis.

Several limitations should be considered. First, the review was restricted to English-language, peer-reviewed, full-text studies and did not include grey literature. This may have introduced language and selection bias and may have excluded relevant evidence from non-English settings, unpublished implementation reports, dissertations, conference proceedings, or organizational documents. Therefore, the findings should be interpreted as a synthesis of the published peer-reviewed literature rather than a complete account of all digital diabetes care initiatives involving nurses. Second, the included studies were heterogeneous in design, setting, digital modality, and outcome reporting. As a result, the review was able to identify recurring patterns and themes, but not to compare findings in a standardized way across studies. Third, some studies contributed more strongly to understanding implementation and service delivery than to direct patient outcomes, which reflects the mixed and still developing nature of this literature. Fourth, digital health literacy was rarely measured explicitly and was more often reflected indirectly through concepts such as supported use, digital confidence, portal engagement, or help with navigating technology. This limits how precisely the review can address digital health literacy as a distinct outcome. Finally, although a methodological quality appraisal was added to support interpretation, the appraisal was used to contextualize the evidence rather than to exclude studies. The included studies remained heterogeneous in design, setting, sample size, digital modality, outcome measurement, and reporting of nursing tasks. Therefore, the findings should be interpreted as a structured map and critical synthesis of the evidence rather than as a pooled estimate of effectiveness. No patient or caregiver partners were directly involved in formulating the review question, interpreting the themes, or preparing the manuscript. Because this review focused on published literature, patient perspectives were included only indirectly through the primary studies that reported patient experiences. Future reviews and intervention studies should consider direct patient or caregiver involvement to strengthen the relevance and interpretation of findings.

## 5. Conclusions

This scoping review mapped how nurses were described in the literature as supporting adults with type 2 diabetes in using digital tools, information, and services for self-management across care settings. Across the included studies, nurses commonly contributed through patient education, remote monitoring, care coordination, digital onboarding, interpretation of digital feedback, implementation support, and equity-focused assistance for patients with limited access, literacy, or digital confidence. These findings suggest that nursing support may be an important component of digitally supported diabetes care, particularly when digital tools require patient education, follow-up, interpretation, or workflow integration.

For nursing practice and service implementation, the findings highlight several areas that may warrant attention, including role definition, training in digital communication and telemonitoring, time for onboarding and follow-up, escalation pathways for abnormal readings or patient concerns, workload planning, staffing, workflow integration, and equity-focused support. Future research could measure digital health literacy and digital engagement more consistently, report nursing tasks in greater detail, and examine how nurse-supported digital care is implemented across diverse patient populations and care settings.

## Figures and Tables

**Table 1:** Characteristics of included studies examining nurses’ roles in supporting digital engagement and self-management in adults with type 2 diabetes.

Study	Country/Setting	Study Design and Sample	Digital Support/Intervention	Main Nursing Role
Jakobsen et al. (2021) [12]	Denmark; four general practices	Qualitative feasibility study; general practitioners and practice nurses in four general practices	Digital individualized coaching and treatment support linked to general practice	Practice nurses supported integration of the digital coaching model into routine care and contributed to follow-up and feasibility assessment
Kassavou et al. (2021) [21]	United Kingdom; eight primary care practices	Mixed-method process evaluation; 57 intervention cases in quantitative analyses, plus interviews with 20 patients and 8 practice nurses	Highly tailored digital intervention to support medication adherence in primary care	Practice nurses contributed implementation feedback and helped clarify how digital adherence support could be incorporated into routine primary care
Roca et al. (2021) [3]	Spain; primary care setting	Nine-month pilot study; 13 adults with type 2 diabetes and depressive disorder, plus 5 nurses	Virtual assistant delivered through the Signal messaging platform	Nurses monitored patients through the platform and evaluated the usefulness of the assistant in practice
Crowley et al. (2022) [2]	United States; two Veterans Affairs health care systems	Randomized clinical trial; 200 adults with persistently poor type 2 diabetes control	Nurse-delivered telehealth including telemonitoring, self-management support, diet/activity support, medication management, and depression support	Nurses acted as telehealth educators, remote monitors, medication support providers, and care coordinators
Ngassa Piotie et al. (2022) [15]	South Africa; primary care services in Tshwane District	Qualitative SWOT analysis; online discussion with 4 field researchers and 3 managers, plus interviews with 7 nurses, 5 doctors, and 13 patients	Tshwane Insulin Project with app-enabled insulin initiation/titration, glucose-monitoring devices, community follow-up, and telehealth support	Professional nurses assessed patients, initiated insulin, provided education, and worked within a telehealth-enabled diabetes support model
Tan et al. (2022) [4]	China; diabetes specialist service	Prospective study; 464 adults with type 2 diabetes	Doctor–nurse–patient mobile health management system with education, hotline access, reminders, and scheduled follow-up	Nurses delivered diabetes education, arranged follow-up, entered glucose-related information, and supported use of the mobile system
Zamanillo-Campos et al. (2022) [22]	Spain; primary care in Mallorca	Qualitative study with focus groups and interviews; 28 primary care professionals in focus groups and 8 interview participants	DiabeText mHealth text-message intervention	Nurses contributed to content tailoring, feasibility planning, and consideration of workflow fit in routine primary care
Butler et al. (2023) [11]	United Kingdom; general practice settings	Qualitative study; 46 general practice staff across 7 focus groups and 5 interviews	SuMMiT-D text message intervention for medication adherence	Nurses contributed to implementation planning, recruitment pathways, workflow assessment, and practice-level support
Jarl et al. (2023) [23]	Sweden; primary health care	Qualitative focus group study; 14 adults with type 2 diabetes and 4 diabetes specialist nurses, plus field notes from a meeting with 18 diabetes specialist nurses	Planned digital intervention for diabetes self-management education and support (DSMES)	Diabetes specialist nurses were positioned as educators, providers of personalized feedback, and partners in designing digital DSMES
Tan et al. (2024) [24]	Singapore; public primary care clinic	Qualitative study with semistructured interviews; 21 adults who completed 6 months of telemonitoring	Multicomponent telemonitoring system with immediate feedback, educational videos, automated reminders, and threshold-triggered nurse support	Telemonitoring nurses responded to abnormal readings, reinforced self-care, and delivered personalized remote support
Hoo et al. (2025) [25]	Malaysia; Malaysian JADE diabetes program	Long-term prospective follow-up of a randomized multicomponent program; 1196 adults at baseline, 826 returned for repeat assessment	Web-based JADE platform with structured risk assessment, personalized reports, and nurse telephone follow-up	Nurses collected structured assessment data, reinforced use of personalized reports, and provided telephone follow-up
Li et al. (2025) [13]	China; multicenter program across 574 hospitals	Prospective observational real-world study; 199,431 adults initiating basal insulin therapy, 118,134 completed 3-month follow-up	TRIO optimal health management program with app-enabled data sharing, WeChat support, health education, and phone follow-up	Nurses delivered education, psychological support, phone follow-up, and review of uploaded or self-reported glucose data
Whittemore et al. (2025) [5]	United States; two community health centers	Single-arm pre-post pilot study; 23 adults with type 2 diabetes who were portal-naive at baseline	Multilevel patient portal intervention with tablet/cell service, individualized portal training, and nurse portal communication	A nurse met individually with participants to create a diabetes self-management support plan and communicated through the portal over 6 months
Xin et al. (2026) [16]	China; single-hospital follow-up program	Retrospective cohort study; 280 adults with type 2 diabetes (100 conventional management; 180 personalized management)	Artificial intelligence and big data-driven personalized chronic disease management app	Specialized nurses delivered group education and one-to-one explanations; the digital model was evaluated against conventional nurse-led management
van den Berg et al. (2026) [14]	Netherlands; five Dutch general practices	Qualitative implementation study with supplementary patient quantitative data; 3 general practitioners, 8 specialized practice nurses, 5 patients, and 4 care-group stakeholders	MiGuide mobile health app and online lifestyle platform integrated into primary care	Specialized practice nurses were central users and implementation partners in app-supported diabetes care

**Table 2:** Thematic synthesis of nurses’ roles in supporting digital engagement and self-management in adults with type 2 diabetes.

Theme	How the Role Appeared	Representative Studies	Interpretive Note
Nurses as digital self-management educators	Nurses helped patients understand diabetes care tasks and use the digital component itself, including glucose monitoring, insulin routines, portal communication, app-supported learning, and self-management reinforcement.	[2,4,5,13,15,22]	Educational work was usually ongoing and blended with monitoring, reminders, and motivational reinforcement rather than delivered as a one-time teaching event.
Nurses as remote monitors and care coordinators	Nurses reviewed transmitted data, responded to threshold-triggered alerts, reinforced treatment plans, coordinated follow-up, and linked digital information to clinical decision making.	[2,3,4,13,15,21,22]	This role was especially visible in telemonitoring, insulin-support, and glucose-management models where patients required repeated contact between visits.
Nurses as facilitators of digital engagement, confidence, and supported use	Nurses helped patients start using digital tools, navigate portals or apps, interpret messages or feedback, and sustain participation over time.	[3,4,5,14,22,25]	Whittemore et al. was the only study to directly measure digital health literacy; the remaining studies addressed supported use, confidence, or interpretation more indirectly.
Nurses as implementation partners in digital diabetes care	Nurses shaped how digital interventions were introduced, tailored, embedded, and sustained within primary care, specialty care, and large-scale service models.	[11,12,14,15,20,24,25]	Implementation success depended on workflow fit, onboarding processes, technical support, staffing capacity, and clear responsibilities.
Equity, access, and context shape digital diabetes support	Digital diabetes support was influenced by age, digital confidence, health literacy, language, culture, socioeconomic context, connectivity, and local infrastructure.	[3,4,5,14,15,16,25]	Several studies warned that digital programs may exclude patients with low digital confidence, limited literacy, weak connectivity, or fewer material resources unless support is intentionally built in.

## Data Availability

Data extracted and synthesized for this review are available in the article and Supplementary Materials.

## Supplementary Materials

The following supporting information can be downloaded at: https://www.mdpi.com/article/10.3390/nursrep16060191/s1, Table S1: Electronic search strategies and database yields; Table S2: Summary of methodological quality appraisal of included studies informed by the Mixed Methods Appraisal Tool; PRISMA-ScR Checklist.

## Public Involvement Statement

No patients, caregivers, or members of the public were directly involved in the design, conduct, interpretation, or writing of this scoping review.

## Guidelines and Standards Statement

This manuscript was prepared in accordance with the Preferred Reporting Items for Systematic Reviews and Meta-Analyses Extension for Scoping Reviews (PRISMA-ScR).
