# Supplementary material for: Nurses’ Roles in Supporting Digital Engagement and Self-Management in Adults with Type 2 Diabetes: A Scoping Review
Source: Nurs Rep. 2026 Jun 4;16(6):191. doi: 10.3390/nursrep16060191 (PMC13304534; doi:10.3390/nursrep16060191)
Supplement: Supplementary file 1 [file nursrep-16-00191-s001.zip › Supplementary Table S1.pdf]

Supplementary Table S1. Electronic search strategies and database yields

Database	Search date	Search strategy	Records identified
**PubMed**	Jan-26	(type 2 diabetes OR T2DM) AND (nurs* OR nurse-led OR nursing intervention* OR diabetes nurse* OR nurse educator*) AND (digital health literacy OR eHealth literacy OR electronic health literacy OR mHealth literacy OR digital literacy OR telehealth OR telemedicine OR mHealth OR eHealth OR mobile app* OR patient portal* OR online health information OR web-based) AND (self-management OR self care)	58
**Scopus**	Jan-26	TITLE-ABS-KEY (("type 2 diabetes" OR T2DM) AND (nurs* OR "nurse-led" OR "nursing intervention*" OR "diabetes nurse*" OR "nurse educator*") AND ("digital health literacy" OR "eHealth literacy" OR "electronic health literacy" OR "mHealth literacy" OR "digital literacy" OR telehealth OR telemedicine OR mHealth OR eHealth OR "mobile app*" OR "patient portal*" OR "online health information" OR "web-based") AND ("self-management" OR "self care")) AND PUBYEAR > 2020 AND PUBYEAR < 2027	69

**Embase**	Jan-26	(('type 2 diabetes' OR T2DM):ti,ab,kw) AND ((nurs* OR 'nurse-led' OR 'nursing intervention*' OR 'diabetes nurse*' OR 'nurse educator*'):ti,ab,kw) AND (('digital health literacy' OR 'ehealth literacy' OR 'electronic health literacy' OR 'mhealth literacy' OR 'digital literacy' OR telehealth OR telemedicine OR mhealth OR ehealth OR 'mobile app*' OR 'patient portal*' OR 'online health information' OR 'web-based'):ti,ab,kw) AND (('self-management' OR 'self care'):ti,ab,kw) AND [2021-2026]/py	99
**EBSCO/CINAHL**	Jan-26	TX ("type 2 diabetes" OR T2DM) AND TX (nurs* OR "nurse-led" OR "nursing intervention*" OR "diabetes nurse*" OR "nurse educator*") AND TX ("digital health literacy" OR "eHealth literacy" OR "electronic health literacy" OR "mHealth literacy" OR "digital literacy" OR telehealth OR telemedicine OR mHealth OR eHealth OR "mobile app*" OR "patient portal*" OR "online health information" OR "web-based") AND TX ("self-management" OR "self care")	4

Note: Search strategies were adapted to the syntax, field structure, and indexing functions of each database. Publication-year eligibility was limited to studies published from 2021 to 2026. Where the publication-year limit was not embedded directly in the database search string, it was applied during eligibility screening. The total number of records identified across all databases before deduplication was 230.
