# Supplementary material for: Nurses’ Roles in Supporting Digital Engagement and Self-Management in Adults with Type 2 Diabetes: A Scoping Review
Source: Nurs Rep. 2026 Jun 4;16(6):191. doi: 10.3390/nursrep16060191 (PMC13304534; doi:10.3390/nursrep16060191)
Supplement: Supplementary file 1 [file nursrep-16-00191-s001.zip › Supplementary Table S2.pdf]

Supplementary Table S2. Summary of methodological quality appraisal of included studies informed by the Mixed Methods Appraisal Tool (MMAT)

Study	Study Design	MMAT Category	Key Strengths	Key Limitations	Overall Evidence Weight
Jakobsen et al. (2021)	Qualitative feasibility study in four general practices	Qualitative	Clear feasibility focus; relevant perspectives from general practitioners and practice nurses; useful insight into role distribution, referral processes, and workflow fit in general practice	Small number of practices; feasibility-focused rather than outcome-focused; limited patient-level effectiveness data; findings may be context-specific to Danish general practice	Moderate
Kassavou et al. (2021)	Mixed-methods process evaluation of a digital medication adherence intervention	Mixed methods	Combined quantitative process data, digital logfiles, patient interviews, and practice nurse interviews; useful for understanding behavior-change mechanisms and implementation conditions	Process evaluation rather than primary effectiveness study; relatively small complete intervention sample; included hypertension and/or type 2 diabetes, so diabetes-specific nursing implications require cautious interpretation	Moderate
Roca et al. (2021)	Nine-month pilot validation study with quantitative outcomes and participant interviews	Mixed methods	Direct testing of a virtual assistant integrated into a messaging platform; included both patient and nurse evaluation; measured medication adherence and clinical/psychological indicators	Very small sample; no control group; pilot design limits causal inference; focused on patients with comorbid type 2 diabetes and depressive disorder, which may limit generalizability	Low–Moderate

Crowley et al. (2022)	Active-comparator randomized clinical trial	Quantitative randomized controlled trial	Strong randomized design; clearly defined intervention arms; nurse-delivered telehealth intervention; clinically meaningful outcomes including HbA1c, self-care, self-efficacy, distress, adverse events, and cost	Conducted in two Veterans Affairs systems; sample may not generalize to broader populations; intervention required infrastructure and staffing resources; some outcomes may be context-dependent	High
Ngassa Piotie et al. (2022)	Qualitative SWOT analysis of a complex insulin-management intervention	Qualitative	Included multiple stakeholder perspectives, including nurses, doctors, managers/researchers, and patients; strong implementation relevance in resource-constrained primary care; clearly identified barriers and facilitators	Small qualitative sample; focused on implementation conditions rather than patient outcome effectiveness; findings are specific to the South African primary care context	Moderate
Tan et al. (2022)	Prospective study of a doctor–nurse–patient mobile health management system	Quantitative non-randomized	Larger prospective sample; objective glycemic outcome using fasting plasma glucose; structured follow-up through the mobile health management system; age-stratified analysis	No randomized comparison group; short follow-up period; potential selection and adherence bias; limited detail on nursing workload and fidelity of intervention delivery	Moderate
Zamanillo-Campos et al. (2022)	Qualitative study with focus groups and interviews of primary care professionals	Qualitative	Clear qualitative design; included multiple primary care professionals; useful implementation insight into acceptability, tailoring, workflow, and feasibility of a text-message intervention	Professional perspectives only; no patient outcome data; intervention was under development rather than fully implemented; limited inference about effectiveness	Moderate

Butler et al. (2023)	Qualitative study with general practice staff	Qualitative	Large qualitative sample for implementation work; included multidisciplinary primary care staff; strong insight into responsibilities, resources, workflow compatibility, and implementation priorities	Did not directly measure patient outcomes; focused on staff perspectives; nursing-specific findings are embedded within wider general practice staff data	Moderate
Jarl et al. (2023)	Qualitative focus group study with patients and diabetes specialist nurses	Qualitative	Included both patient and diabetes specialist nurse perspectives; directly relevant to digital diabetes self-management education and support; identified practical needs for information, feedback, and integration into routine care	Small sample; focused on anticipated needs for digital intervention rather than tested implementation; limited ability to assess effectiveness or sustained engagement	Moderate
Tan et al. (2024)	Qualitative study of adults completing a telemonitoring intervention	Qualitative	Theory-informed analysis using the Health Belief Model; direct patient experience after six months of telemonitoring; clear relevance to self-efficacy, feedback, reminders, and nurse-supported behavior change	Included only participants who completed telemonitoring, creating possible selection bias; did not include nurse interview data; limited ability to infer effectiveness beyond participant-reported experience	Moderate
Hoo et al. (2025)	Seven-year prospective follow-up of a randomized multicomponent JADE program	Quantitative randomized controlled trial / long-term follow-up	Large baseline cohort; long follow-up period; randomized intervention arms; clinically meaningful outcomes including ABC target attainment and diabetes-related endpoints; adjusted	Substantial attrition over seven years; long-term follow-up may be influenced by post-intervention care and survivorship; nursing contribution was part of a	Moderate–High

			analyses used to address follow-up bias	multicomponent program rather than isolated	
Li et al. (2025)	Prospective observational real-world study across 574 hospitals	Quantitative non-randomized	Very large multicenter sample; real-world implementation context; objective clinical outcomes including HbA1c and hypoglycemia; directly relevant to nurse-supported app-enabled insulin initiation and follow-up	No randomized control group; short three-month follow-up; high loss/noncompletion between enrollment and follow-up; limited detail on intervention fidelity and variation in nursing delivery across hospitals	Moderate–High
Whittemore et al. (2025)	Single-arm pre–post pilot study in two community health centers	Quantitative non-randomized	Direct focus on portal-naive adults with type 2 diabetes; measured portal use, technology confidence, digital health literacy, self-efficacy, and distress; strong equity relevance in underserved settings	Small sample; no control group; pilot design limits effectiveness inference; short follow-up; feasibility and acceptability findings stronger than clinical outcome evidence	Low–Moderate
Xin et al. (2026)	Retrospective cohort study comparing personalized AI-supported management with conventional nurse-led management	Quantitative non-randomized	Comparator group included; assessed glycemic control, self-care, and quality of life; six-month follow-up; relevant to AI-supported chronic disease management and nursing education/explanation roles	Retrospective single-hospital design; potential confounding and selection bias; limited generalizability; intervention effects may reflect multiple components beyond nursing support alone	Moderate

van den Berg et al. (2026)	Qualitative implementation study supplemented with quantitative patient data	Mixed methods	Multiple stakeholder groups, including general practitioners, specialized practice nurses, patients, and care-group stakeholders; longitudinal implementation perspective; strong insight into barriers, facilitators, usability, workflow, and context-specific strategies	Very small patient sample for quantitative data; incomplete follow-up data for some outcomes; implementation-focused rather than effectiveness-focused; findings specific to Dutch primary care and MiGuide platform	Moderate

Note: Evidence weight reflects the relative contribution of each study to this scoping review's synthesis, considering study design, sample size, methodological transparency, outcome relevance, and limitations. It is not intended as a numerical MMAT score.
